# Whole-Genome Sequencing Enables Molecular Characterization of Non-Clonal Group 258 High-Risk Clones (ST13, ST17, ST147 and ST307) among Carbapenem-Resistant *Klebsiella pneumoniae* from a Tertiary University Hospital Centre in Portugal

**DOI:** 10.3390/microorganisms10020416

**Published:** 2022-02-11

**Authors:** Gabriel Mendes, João F. Ramalho, Ana Bruschy-Fonseca, Luís Lito, Aida Duarte, José Melo-Cristino, Cátia Caneiras

**Affiliations:** 1 Laboratório de Investigação em Microbiologia na Saúde Ambiental (EnviHealthMicro Lab), Instituto de Saúde Ambiental (ISAMB), Faculdade de Medicina, Universidade de Lisboa (ULisboa), 1649-026 Lisboa, Portugal; gabriel-mendes@campus.ul.pt (G.M.); jfrancisko.ramalho@gmail.com (J.F.R.); 2 Laboratório de Microbiologia, Serviço de Patologia Clínica, Centro Hospitalar Universitário Lisboa Norte, 1649-035 Lisboa, Portugal; ana.bruschy@chln.min-saude.pt (A.B.-F.); lmlito@chln.min-saude.pt (L.L.); melo_cristino@medicina.ulisboa.pt (J.M.-C.); 3 Faculdade de Farmácia, Universidade de Lisboa, 1649-033 Lisboa, Portugal; aduarte@ff.ulisboa.pt; 4 Centro de Investigação Interdisciplinar Egas Moniz, Instituto Universitário Egas Moniz, 2829-511 Monte da Caparica, Portugal; 5 Instituto de Microbiologia, Faculdade de Medicina, Universidade de Lisboa (ULisboa), 1649-028 Lisboa, Portugal; 6 Instituto de Medicina Preventiva e Saúde Pública (IMP&SP), Faculdade de Medicina, Universidade de Lisboa (ULisboa), 1649-026 Lisboa, Portugal

**Keywords:** *Klebsiella pneumoniae*, KPC-3, OXA-181, NDM-1, whole-genome sequencing (WGS), carbapenem-resistance, molecular epidemiology, hypermucoviscosity (HMV), hypervirulent, ST13, ST17, ST307, ST147, ST39-KL62, Portugal

## Abstract

The carbapenem-resistant Enterobacterales (CRE) strains have been identified by the World Health Organization as critical priority pathogens in research and development of diagnostics, treatments, and vaccines. However, recent molecular information about carbapenem-resistant *K. pneumoniae* (CRK) epidemiology in Portugal is still scarce. Thus, this study aimed to provide the molecular epidemiology, resistome, and virulome of CRK clinical strains recovered from a tertiary care hospital centre (2019–2021) using polymerase chain reaction (PCR) and the advanced molecular technique whole-genome sequencing (WGS). PCR amplification of carbapenemase genes was performed in 437 carbapenem-resistant *K. pneumoniae* strains. The most frequent carbapenemases were: KPC-3 (42%), followed by OXA-181 (20%), GES-5 (0.2%), and NDM-1 (0.2%). Additionally, 10 strains (2%) coproduced KPC-3 and OXA-181, and 1 strain coproduced KPC-3 and OXA-48 (0.2%). The genomic population structure of 68 strains characterized by WGS demonstrated the ongoing dissemination of four main high-risk clones: ST13, ST17, ST147, and ST307, while no clones belonging to the European predominant clonal groups (CG15 and CG258) were found. Moreover, we describe one *K. pneumoniae* ST39-KL62 that coproduced the NDM-1 carbapenemase and the extended-spectrum beta-lactamase CTX-M-15, and one *K. pneumoniae* ST29-KL54 GES-5 and BEL-1 coproducer. Furthermore, a high prevalence of iron siderophores were present in all CRK strains, with several strains presenting both colibactin and the hypermucoviscosity phenotype. Thus, the data presented here highlight an uncommon molecular epidemiology pattern in Portugal when compared with most European countries, further supporting the emergence and dissemination of nonclonal group 258 hypervirulent multidrug high-risk clones and the need to promote in-depth hospital molecular surveillance studies.

## 1. Introduction

*Klebsiella pneumoniae* is a Gram-negative bacterium with a high public health importance, capable of causing severe organ infections and life-threatening disease in both community- and hospital-acquired infections. *K. pneumoniae* has emerged as an increasingly resistant pathogen due to its predisposition to acquire multidrug and carbapenem resistance, limiting the therapeutic options [1]. A large variety of carbapenemases have been identified worldwide in *K. pneumoniae*, namely KPC, GES (Ambler class A), NDM, VIM, IMP (class B), and OXA (Class D), among others [2], positioning *K. pneumoniae* as one of the top three pathogens of international concern, according to the World Health Organization’s Global Priority List of Antibiotic-Resistant Bacteria to Guide Research, Discovery, and Development of New Antibiotics [3].

In Portugal, after the isolation of the first carbapenemase producing a clinical strain of *K. pneumoniae* at a Lisbon hospital in 2009 [4], an increasing trend of carbapenem resistance among *K. pneumoniae* has been described, evolving from 3.4% in 2015 to 10.9% in 2019, as reported by the European Centre for Disease Prevention and Control (ECDC) [5]. The last available data in Portugal showed that the most frequent carbapenemase produced *K. pneumoniae* there were KPC-3 followed by OXA-181 [6,7,8], although other carbapenemases such as GES-5 and OXA-48 were also reported [9,10]. Despite its presence in several countries in Europe, as well as its worldwide spread, the NDM carbapenemase is not frequent in Portuguese clinical strains [11].

*K. pneumoniae* utilizes a variety of virulence factors such as capsule polysaccharides, adhesins, fimbriae, and iron-binding siderophores (aerobactin, enterobactin, salmochelin, and yersiniabactin) for survival and immune evasion during infection. Although the siderophore content and expression may be variable among different *K. pneumoniae* genetic lineages, the ability to sequester iron seems to contribute to the pathogenic potential of this organism [1]. Of relevance, it was identified that multidrug-resistant sequence type ST14 KPC-3 carbapenemase *K. pneumoniae* strains presented a high prevalence of virulent determinants, including the K2 capsular antigen and the siderophore aerobactin [12].

Previous studies indicated that molecular epidemiology in Portugal seems different from the existing molecular epidemiology in Europe. The most common clones detected in the European region are ST11, ST258, and ST512, all belonging to clonal group 258 (CG-258) [13]. In contrast, the most dominant STs reported so far in Portugal are ST13, ST14, ST15, ST17, ST147, ST307 and ST348 [6,7,8,9,12,14,15], all presenting high potential for virulence and dissemination. However, recent data on genomic surveillance of healthcare-associated infections by *K. pneumoniae* in Portugal are still limited, and the most recent study included strains recovered until 2019 [10]. Moreover, despite the recent interest in the interplay between resistance and virulence determinants in *K. pneumoniae* clinical strains, their interplay in these uncommon clones remains poorly understood [16]. Therefore, the aim of the present study was to provide updated information on the genomic epidemiology, resistome, and virulome of healthcare-associated infections by *K. pneumoniae* strains identified at a tertiary university hospital centre in Portugal (2019–2021).

## 2. Materials and Methods

### 2.1. Bacterial Strains

A total of 437 carbapenem-resistant *K. pneumoniae* clinical strains were recovered from a tertiary university hospital centre in Lisbon over a 2-year period, September 2019–2021, using standard clinical operating procedures, and were sent to the Laboratory of Microbiology Research in Environmental Health of the Faculty of Medicine, Universidade de Lisboa (ULisboa), for advanced molecular analysis. The identification was performed by microbiology laboratories using conventional methods or automated systems such as Vitek2^®^ (BioMérieux, Marcy, l’Étoile, France) or MicroScan (Snap-on, Kenosha, WI, USA). All strains were maintained frozen in BHI broth (VWR Prolabo, Lisbon, Portugal) plus 15% glycerol at −80 °C. For analysis, the strains were grown in BHI broth for 18 h at 37 °C and seeded in Mueller Hinton agar (VWR Prolabo, Lisbon, Portugal).

### 2.2. Antimicrobial Susceptibility Testing

The standardized Kirby–Bauer disk diffusion technique was performed for antimicrobial susceptibility testing, in accordance with the European Committee on Antimicrobial Susceptibility Testing (EUCAST) guidelines. The detailed methodology is available at http://www.eucast.org/ast_of_bacteria/disk_diffusion_methodology (accessed on 20 December 2021). Detailed instructions for Mueller–Hinton agar medium (VWR Prolabo, Lisbon, Portugal), including preparation and storage, are also available in the same EUCAST guidelines document. Susceptibility was tested for a panel of antibiotics: amoxicillin/clavulanic acid (20/10 µg), cefoxitin (30 µg), cefotaxime (5 µg), ceftazidime (10 µg), imipenem (10 µg), gentamicin (10 µg), ciprofloxacin (5 µg), tigecycline (15 µg), aztreonam (30 µg), ertapenem (10 µg), meropenem (10 µg), and ceftazidime–avibactam (10/4 µg) (Biorad, Algés, Portugal). The strains were categorized as susceptible, standard dosing regimen (S); susceptible, increased exposure (I); and resistant (R) by applying the breakpoints in the phenotypic test results. The inhibition zones were interpreted according to the EUCAST breakpoint tables for interpretation of minimum inhibitory concentration (MIC) and zone diameters (version 11.0, 2021, available at https://eucast.org/clinical_breakpoints; accessed on 20 December 2021). Multidrug-resistant (MDR) bacteria were defined as those that acquired nonsusceptibility to at least one agent in three or more antimicrobial categories, in accordance with the United States Centers for Disease Control and Prevention (CDC) and the European Centre for Disease Prevention and Control (ECDC) consensual definition [17].

### 2.3. Hypermucoviscosity Phenotype

The string test was performed to access the hypermucoviscosity (HMV) phenotype of the strains. A positive string test was defined as the formation of viscous strings of >5 mm in length when a loop was used to stretch the colony on an agar plate.

### 2.4. Molecular Methods of Species Identification and Detection of Carbapenemase Genes

PCR-based screening was performed for confirm the *K. pneumoniae* identification using the universal 16S rRNA bacterial primers 27F F:5′-AGAGTTTGATCCTGGCTCAG-3′; 1392R R:5′- GGTTACCTTGTTACGACTT -3′ and to identify carbapenemases genes with primers designed for *bla*_OXA-48_ F:5′-GGCTGTGTTTTTGGTGGCATC-3′; R:5′-GCAGCCCTAAACCATCCGATG-3′, *bla*_KPC_ [18], *bla*_VIM_ [19], *bla*_NDM_ [20], *bla*_GES_ [21]) using the NZYTaq II 2× Green Master Mix (NZYTech, Lisbon, Portugal) following the manufacturer’s instructions. The PCR products were resolved in 1% agarose gel in 10× concentrated Tris-Borate-EDTA (TBE buffer) (NZYTech, Lisbon, Portugal). The PCR assays included positive and negative controls. The positive controls used were positive strains previously sequenced by the Sanger sequencing method. These positive controls were submitted to purification using an ExoCleanUp FAST kit (VWR Prolabo, Lisbon, Portugal) and were sequenced by the Sanger sequencing method at STABVida, Caparica, Portugal. Searches for nucleotide sequences were performed with the BLAST program, which is available at the National Center for Biotechnology Information website (http://www.ncbi.nim.nih.gov; accessed on 20 December 2021). Multiple-sequence alignments were performed with the Clustal Omega program, which is available at https://cge.cbs.dtu.dk/services (accessed on 20 December 2021).

### 2.5. Whole-Genome Sequencing (WGS)

We performed WGS on carbapenem-resistant *K. pneumoniae* strains with the objective of gaining further insight on the molecular epidemiology, resistome, and virulome of these strains. A total of 68 nonduplicated *K. pneumoniae* clinical strains were selected based on the carbapenemase gene type identified and within these, by random selection when the number of strains was above one, as previously carried out [6]. The genomic DNA were extracted for WGS from cultures grown overnight in Mueller–Hinton agar, using the NZY Tissue gDNA Isolation kit (NZYTech, Lisbon, Portugal), as per the manufacturer’s recommendations. The Sequence was done at STABVida Portugal. Sequencing libraries were prepared using the KAPA HyperPrep Library Preparation Kit (Roche, Basel, Switzerland) following the manufacturer’s recommended protocol, and sequenced using an Illumina HiSeq Novaseq 6000 platform (Illumina, San Diego, CA, USA) with paired-end reads (2 × 151 bp). The raw data quality control was performed using FASTQC v0.11.9 (Babraham Institute, Cambridge, UK) and the trimming and *de novo* assembly were performed using CLC Genomics Workbench 12.0.3 (QIAGEN, Aarhus, Denmark). All assemblies were carried out with automatic word size, similarity fraction of 0.95, a length fraction of 0.95, and a minimum contig size of 500 bp.

### 2.6. Drug-Resistance-Associated Genes, Virulence Genes, Capsular Types, and Plasmid Replicons

Antimicrobial resistance (AMR), virulence, K and O antigen loci, and the Multilocus Sequence Typing (MLST) were identified using Kleborate, a genomic typing tool specific to *K. pneumoniae* available at https://github.com/katholt/Kleborate (accessed on 6 October 2021). Plasmid analyses were identified using the PlasmidFinder database (https://cge.cbs.dtu.dk/services/PlasmidFinder; accessed on 6 October 2021), with the following cut-off values: minimum of 60% coverage and 95% identity.

### 2.7. Phylogenetic Analysis

A minimum-spanning tree (MST) was constructed using the Phyloviz V2.0 software (available at https://bitbucket.org/phyloviz/phyloviz-main/downloads; accessed on 6 October 2021) and the goeBURST algorithm therein implemented. The MLST data used in the construction of the MST was retrieved from the downloadable scheme MLST profiles of *K. pneumoniae* available at the Pasteur MLST website (http://www.pasteur.fr/mlst; accessed on 6 October 2021).

### 2.8. Ethical Approval

The strains were obtained as part of routine diagnostic testing and were analysed anonymously. The study proposal was analysed and dismissed from evaluation by the Ethics Committee of the Lisbon Academic Medical Centre of the Faculty of Medicine, Universidade de Lisboa, Portugal (Nr. 248/21, 22 September 2021).

## 3. Results

### 3.1. Antimicrobial Susceptibility and HMV Phenotype

The antimicrobial susceptibility test showed lower susceptibility rates to amoxicillin/clavulanic acid (2/437; 0.5%), ertapenem (27/437; 3.9%), ceftazidime and aztreonam (both 33/437; 7.6%), cefotaxime (39/437; 8.9%), cefoxitin (97/437; 22.2), gentamicin (113/437; 25.9%), imipenem (122/437; 27.9%), ciprofloxacin (131/437; 30.0%), and meropenem (170/437; 38.9%). Moreover, high susceptibility rates to ceftazidime/avibactam (1/437; 99.8%), and to tigecycline (306/437; 70.0%) were found (Table 1). The HMV phenotype performed by the string test was positive for 11 strains (0.2%).

### 3.2. Identification of Carbapenemase Genes

All strains were confirmed as *K. pneumoniae* according to the 16S rRNA bacterial molecular methodology. PCR amplification of carbapenemase genes was performed in 437 *K. pneumoniae* strains. We identified *bla*_KPC-3_ (182/437; 41.6%), *bla*_OXA-181_ (89/437; 20.4%), *bla*_GES-5_ (1/437; 0.2%), and *bla*_NDM-1_ (1/437; 0.2%), but no *bla*_VIM_ gene was detected. Additionally, 10 strains (10/437; 2.3%) coproduced *bla*_KPC-3_ and *bla*_OXA-181_, and 1 strain coproduced *bla*_KPC-3_ and *bla*_OXA-48_ (1/437; 0.2%).

### 3.3. Molecular Epidemiology by WGS

**Figure 1:**
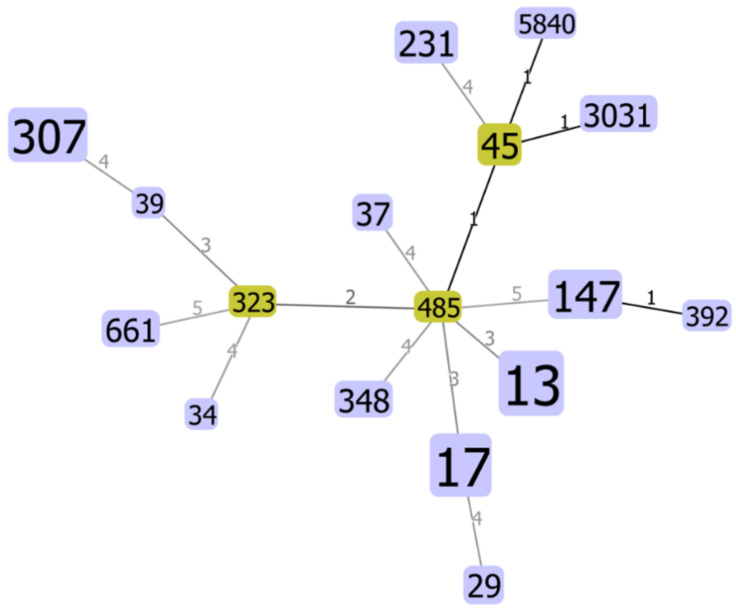
Phylogenetic tree of the STs identified in carbapenem-resistant *K. pneumoniae* clinical strains (*n* = 68), as generated by goeBURST Full MST on PHYLOViZ V2.0. Subgroup founders are highlighted in green. Darker links represents a single-locus variants.

Detailed information about the assembly of the 68 carbapenem-resistant *K. pneumoniae* sequenced by WGS and *de novo* assembled genomes are presented in Table S1. Phylogenetic analysis revealed that the *K. pneumoniae* belonged to 17 different clones (STs). The most predominant clones found were ST13 (17/68; 25.0%), ST17 (15/68; 22.1%), ST307 (8/68; 11.8%), and ST147 (6/68; 8.8%); followed by ST45 (3/68; 4.4%); ST231 (3/68; 4.4%); ST29, ST37, ST348, ST661, and ST3031 (all 2/68; 3.0%); and ST34, ST39, ST485, ST323, ST392, and a novel ST5840 (all 1/68; 1.5%). The phylogenetic tree constructed with the STs found in this study indicated a close relationship between ST485 and ST45, ST147 and ST392, and between ST45 and both ST3031 and the novel ST5840 (Figure 1). These pairs were single-locus variants (SLV), which means they differed from each other in one housekeeping gene allele.

**Figure 2:**
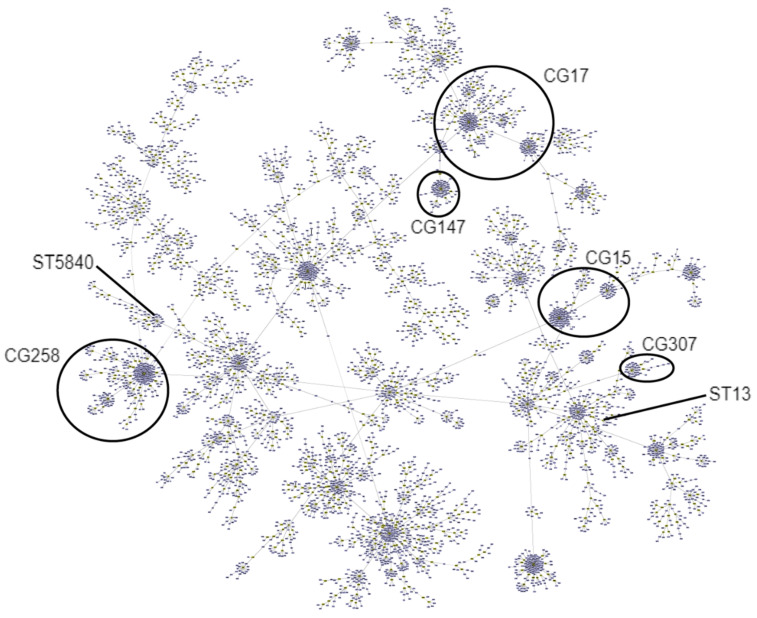
Phylogenetic tree of all worldwide *K. pneumoniae* allelic profiles available at the Pasteur MLST website. The MLST data used in the construction of the minimum-spanning tree was retrieved through the downloadable scheme MLST profiles of *K. pneumoniae* available at the Pasteur MLST website (http://www.pasteur.fr/mlst; accessed on 6 October 2021). Phylogenetic reconstruction was generated by goeBURST Full MST on PHYLOViZ V2.0 and the most common ST found (ST13, ST17, ST147, ST307, and the novel ST5840) are identified. ST17 belongs to CG17, ST147 to CG147, and ST307 to CG307. ST13 and ST5840 do not belong to any of the CGs showed in this phylogenetic tree.

Most ST13 clones were associated with either KPC-3 or KPC-3 and OXA-181/OXA-48 coproducing strains. These strains also showed high predominance in ICEKp10 harboring both *ybt* 17 and clb3 genes, as well as the presence of KL3/KL19, which is associated with O1v2 serotypes. Of note, out of the 17 ST13 clones identified, 5 (29.4%) had an HMV phenotype. Additionally, both ST17 and ST147 clones were associated with OXA-181-producing strains, all of which presented ICEKp12 harboring the *ybt* 16 gene. ST17 was associated with KL25;O5 serotypes, whereas ST147 was mainly associated with KL64;O2v1 serotypes. Moreover, among ST307 clones, one strain (1/8; 12.5%) produced carbapenemase (KPC-3), six strains (6/8; 75%) had OmpK35/OmpK36 porin mutations, and four strains (4/8; 50%) presented an HMV phenotype. Even though previous studies have asserted that CG258 is responsible for most of carbapenem-resistant *K. pneumoniae* infections in Europe [13], none of the STs found belonged to CG258 (Figure 2).

### 3.4. Resistome Characterization

In this collection, 46 strains showed the *bla*_KPC-3_ gene (23/68; 33.8%) and *bla*_OXA-181_ gene (23/68; 33.8%); five strains coproduced the *bla*_KPC-3_ and *bla*_OXA-181_ genes (5/68; 7.4%); and one strain the *bla*_KPC-3_ and *bla*_OXA-48_ genes (1/68; 1.5%). Additionally, two strains showed the *bla*GES-5 gene (1/68; 1.5%) and *bla*_NDM-1_ gene (1/68; 1.5%) (Table 2). Furthermore, we identified two genes coding for extended-spectrum β-lactamases (ESBLs), namely *bla*_CTX-M-15_ and *bla*_BEL-1_; and 10 genes coding for narrow/broad spectrum beta-lactamases (*bla*_TEM-1_, *bla*_OXA-1_, *bla*_OXA-9_, *bla*_OXA-10_, *bla*_SHV-1_, *bla*_SHV-11_, *bla*_SHV-26_, *bla*_SHV-27_, *bla*_SHV-28_, and *bla*_SHV-187_).

Quinolone resistance mechanisms such as *qnrS* and *qnrB* genes were detected in 44 strains (64.7%). The *aac*, *aad*, *aph*, *strA*, and *strB* aminoglycoside-modifying enzyme genes were identified in most (65/68; 95.6%) strains. Trimethoprim–sulfamethoxazole resistance was encoded by *dfr* (*dfrA1*, *dfrA12*, *dfrA14*, *dfrA17*, *dfrA27*) in 64 strains (94.1%) and *sul (sul1* and *sul2*) in 63 (92.6%) strains. The carriage of the *tet(A)*, *tet(D)*, and *tet(J)* gene variant were found in 29 strains (42.6%). Mutations in *gyrA* (A87N, A83I, A87F, A87N) or *parC* (C80I) loci were detected (20/68; 29.4%), as well as mutations in the *OmpK35* (5/68; 7.4%) and *OmpK36* (7/68; 10.3%) genes. One strain had a mutation in both porins genes (1/68; 1.5%). Chloramphenicol acetyl transferase genes (*catA4*, *catII.2*, *cmlA5*, *floR*) were also identified (8/68; 11.8%). The rifampicin adenosine diphosphate (ADP) ribosylating transferase *arr-3* gene was detected in 22 strains (32.4%) and the *arr-2* gene in one strain (1.5%). The *mphA* gene was also identified in 15 strains (22.1%), and the *mphE* and *msrE* in one strain (1.5%).

### 3.5. Virulome Characterization

Regarding the virulence genes found in our strains, we identified 15 capsular locus types (KL3, KL5, KL14, KL19, KL21, KL24, KL25, KL27, KL30, KL51, KL54, KL62, KL64, KL102, and KL153) and eight antigen O locus (O1v1, O1v2, O2v1, O2v2, O3b, O4, O5 and OL101). Additionally, we identified six integrative conjugative elements (*ICEKp3*, *ICEKp4*, *ICEKp5*, *ICEKp10*, *ICEKp11*, and *ICEKp12*), harboring yersiniabactin *ybt 9*, *ybt 10*, *ybt 14*, *ybt 17*, *ybt 15*, and *ybt 16*, respectively. The *ICEKp10* harbored both colibactin (*clb 3*) virulence factor and yersiniabactin *ybt 17*.

### 3.6. Plasmid Replicon Typing

Concerning plasmid replicon types, a total of 23 replicon types were found: ColKP3; IncFIA(HI1), IncFIB(K)(pCAV1099-114), IncR, IncX3, FIA(pBK30683), FII(pBK30683), IncFIB(K), IncFII(K), ColRNAI, IncFIB(pKPHS1), Col(pHAD28), IncM1, IncL, IncFIB(pNDM-Mar), IncFII(Yp), IncFIB(pQil), IncFII(pCoo), IncN, Col440II, IncC, Col156, and Col440I.

## 4. Discussion

Our study provided updated information on the genomic epidemiology, resistome, and virulome of healthcare-associated infections by *K. pneumoniae* strains in Portugal, and revealed a different molecular epidemiology there (ST13, ST17, ST307, ST147) compared to most countries in Europe (CG 258). Moreover, we reported the dominance of KPC-3 and OXA-181 carbapenemases.

One *K. pneumoniae* strain coproduced the Guiana extended-spectrum carbapenemase GES-5 and the extended-spectrum beta-lactamase BEL-1. Both GES-5 and BEL-1 genes seem to be commonly identified in *Pseudomonas aeruginosa*, rather than Enterobacterales [22,23]. The first GES-5-producing Enterobacterales in Portugal (2014) was identified in a *K. pneumoniae* recovered from aquatic environmental samples [22]. The association of *bla*_GES-5_ and *bla*_BEL-1_ genes located on a class 3 integron (In1144) was described in a *K. pneumoniae* ST252 clinical strain isolated in 2009 from northern Portugal. In this class 3 integron, the first gene cassette was the *bla*_IMP-8_ gene [24]. Recently, a study described a *K. pneumoniae* ST147 isolated in 2016 at a tertiary care hospital in Lisbon that produced the *bla*_GES-5_ and *bla*_BEL-1_ genes in combination with the carbapenemase KPC-3, and showed a similar class 3 integron (In4885). Although this integron only harbored the *bla*_GES-5_ and *bla*_BEL-1_ genes, and lacked the *bla*_IMP-8_ gene, both integrons were also located in a ColRNAI plasmid [25]. Another study in Portugal described the clinical isolate *K. pneumoniae* ST29, identified in 2017, coproducing both *bla*_GES-5_ and *bla*_BEL-1_ genes but located in a ColE1 plasmid [9]. Interestingly, our *K. pneumoniae* strain, which was isolated from a bloodstream infection in 2020, also belonged to ST29 and showed decreased susceptibility to imipenem and meropenem, but was resistant to ertapenem and also had a class 3 integron (In257) with three gene cassettes (*bla*_GES-5_, *bla*_BEL-1_, and *aac(6*′*)-Ib4* genes) located in a ColRNAI plasmid. The data herein support that for the successful emergence and dissemination of *bla*_GES-5_ genes, in combination or not with other carbapenemases such as IMP-8 and KPC-3, they are integrated in a class 3 integron and located in a ColRNAI plasmid.

The strain resistant to ceftazidime/avibactam identified was a New Delhi metallo-β-lactamase (NDM) producer that is known to be resistant to ceftazidime–avibactam due to the lack of inhibitory effect of avibactam towards such metallo-β-lactamase producers [26], and is not related to the emergence and dissemination of KPC-3 variants [27].

To date, only few cases of members of Enterobacterales producing NDM-1 have been previously reported in Portugal. Two subsequent NDM-1-producing strains were found in *Morganella morganii* and *Proteus mirabilis* from a single patient [28], one NDM-1-producing *Providencia stuartii* [29], and *Enterobacter* spp. producing NDM-1 strains were also found in water samples in Lis River, in the central region of Portugal [23]. NDM carbapenemases are commonly found in *K. pneumoniae* and *Escherichia coli* and are increasing worldwide, particularly in several European countries, including Spain [30]. Nevertheless, the reports in Portugal are scarce. Recently, an outbreak caused by NDM-1-producing *Klebsiella pneumoniae* ST11-KL105 lineage (in clinical and environmental strains) during the COVID-19 pandemic in Portugal was described [31]. However, herein we report a NDM-1-producing *Klebsiella pneumoniae* ST39 clone, which has been previously reported in other countries harboring the *bla*_NDM-1_ gene [32,33]. Our strain coproduced NDM-1 and CTX-M-15 enzymes, and although NDM producers seemed to be very resistant to most therapeutic regimens [2], the fact that this strain was resistant to all antibiotics tested (including tigecycline) is worrisome and requires further attention.

All the strains coproducing both KPC-3 and OXA-181/OXA-48 showed resistance to carbapenems but susceptibility to ceftazidime/avibactam. OXA-48 *K. pneumoniae* producers are not common in Portugal [6,8,12,25,34], despite its emergence in several Mediterranean countries [35]. The first OXA-48 *K. pneumoniae*-producing strains were found in 10 strains in the northern region of Portugal in 2018 [10] and after that, only one other study reported a *K. pneumoniae* OXA-48-producing strain in the same region of Portugal [8]. This study reported a coproduction of KPC-3 and OXA-48 *K. pneumoniae* strain in a different region of the country, suggesting that although it is not a frequent carbapenemase gene in Portugal, the presence of OXA-48 in *K. pneumoniae* is not limited to a certain region.

The emergence and dissemination of *K. pneumoniae* strains that possess both MDR and hypervirulence is a major concern worldwide. The association between genotypes and hypervirulence phenotype is rather complex, and the differences between hypervirulent and classical strains can often be difficult to access. With the help of whole-genome sequencing (WGS) of *K. pneumoniae* strains, it is now clear that some differences exist in the accessory genome between hypervirulent and classical strains, and that the former contain genes related to increased virulence, such as genes that confer HMV, siderophores, and the genotoxin colibactin, among others [36]. Limited data are available regarding virulence determinants in *K. pneumoniae* in Portugal. One study conducted by Caneiras et al. identified higher accumulation of virulence factors as type 1 and type 3 fimbrial adhesins (*fimH* and *mrkD*), iron siderophore aerobactin (*iucC*), the capsule polysaccharide K2 serotype, and haemolysin (*khe*) in hospital-acquired urinary tract infections by MDR *K. pneumoniae* clinical strains compared with strains recovered from a community setting, which showed lower resistance and virulence [6].

In this study, among the 68 *K. pneumoniae* strains studied by WGS, 63 (93%) presented yersiniabactin harbored in distinct integrative and conjugative element (ICEs), and 16 (25%) of the strains with yersiniabactin also had the genotoxin colibactin (*ICEKp10* encodes colibactin synthetic genes) [37]. This is of relevance, since ICEs are extremely prevalent in hypervirulent *K. pneumoniae* lineages, including in nearly 90% of CG23 and close to 75% of all hypervirulent *K. pneumoniae* (hvKp) strains [37]. Furthermore, the HMV phenotype contributes to a large majority of hvKp strains [37], and our data revealed 11 strains with positive HMV phenotype, all of which coded the yersiniabactin virulence gene.

A prevalence of O1/O2 (47/68; 69%) serotypes was present across the studied strains and was congruent with previous reports in Portugal [8,25]. In contrast, a wide variety of K locus serotypes was found herein, where the KL25 (15/68; 22.1%) associated with O5 serotype was the most dominant. Of relevance, no K1/K2 serotypes were found, contrasting with previous findings by Caneiras et al., which included strains recovered until 2013 [6]. On the other hand, a study conducted in Portugal that included strains recovered after 2013 also found no K1/K2 [8], which corroborated the findings of this study, suggesting the emergence of different serotypes in the country. It is known that K1 and K2 serotypes are the most common capsular types in *K. pneumoniae*, which commonly exhibit an HMV phenotype and are usually associated with hypervirulent strains. However, serotyping alone is not an entirely accurate indicator of hypervirulence, as other serotypes aside from K1 and K2 have also been identified among hvKp strains [36]. In fact, among the most common K locus serotypes related to hypervirulent strains is K1, followed by K2, K5, and K57 [37]. Moreover, in contrast to MDR infections, hypervirulent *K. pneumoniae* infections worldwide are dominated by the same subset of lineages, namely CG23, followed by CG65 (including ST65 and ST375) and CG86, among others to a lesser extent [37]. Noteworthy, one strain belonging to the novel ST5840 clone found in this study was associated with the KL5;O3b serotype, *ybt* 10, ICEKp4, and KPC-3 enzyme. Although ST5840 did not seem to be related to the most common clonal groups associated with hvKp infections, nonetheless, the KL5 serotype with yersiniabactin production suggested a possible potential for hypervirulence in this strain.

A vast array of STs (17 in total) were detected. ST13 was the most prevalent, followed by ST17, ST307, and ST147, accounting for more than two-thirds of all studied strains. All ST13 strains were KPC-3 carbapenemase producers, and moreover, coproduced *bla*_KPC-3_ and *bla*_OXA-181_/*bla*_OXA-48_ genes. ST13 clones have been described in Portugal in KPC-3-producing strains [9]; nevertheless, the coproduction of both *bla*_KPC-3_ and *bla*_OXA-181/OXA-48_ genes in *K. pneumoniae* has not yet been reported in this country. ST17 has already been described harboring KPC [10] and OXA-181 [9] in other studies in Portugal. ST147 clones have also been previously reported, but harboring the *bla*_KPC_ gene [8,14,25], opposite to what was found in this study, in which all ST147 strains produced OXA-181. Still, the strains belonging to the ST147 clone had the same KL64/O2v1 serotype, as previously reported [8,14,25]. All ST307 clones presented the KL102/O2v2 serotype, but only one strain produced carbapenemases genes (*bla*_KPC-3_). However, previous reports described ST307 clones with no carbapenemase produced and with the presence of KL102/O2v2 serotypes. Interestingly, a French report described an outbreak with *K. pneumoniae* KPC-producing ST307 clones, as well as KPC-producing ST147 and ST13 clones from patients who had recently established a link with Portugal [38]. This study also supported an emergent molecular shift in *K. pneumoniae* epidemiology in Portugal, as in Europe, particularly for the appearance of ST147 and ST307 high-risk clones [39], a cause of concern [40], and recently associated with high mortality in COVID-19 patients [41].

Indeed, previous national reports of carbapenemase-producing *K. pneumoniae* strains in Portuguese healthcare facilities already revealed a different molecular epidemiology compared to most countries in Europe. To date, it has been reported that Portugal presented more clones belonging to CG15 than to CG258 [6,7,9,12,15]. However, strains belonging to CG15 that were previously frequently found were not present in this study. In fact, the molecular epidemiological characterization of clinical strains resistant to carbapenems demonstrated a different epidemiological scenario in the last 2 years: ST13, ST17, ST147, and ST307 remained among the most frequently found MLST profiles. Additionally, clones belonging to the CG258 were also not present, which highlighted the different molecular epidemiology in Portugal compared the most of the countries in Europe.

Our study had some limitations, given that the WGS analysis was performed on 68 selected *K. pneumoniae* strains and not on the complete collection of strains. Moreover, we included strains from a single tertiary university hospital centre, which cannot represent the complete epidemiological situation in the country. However, it is important to highlight that is one of the major hospital centres in Portugal, and considering that it is a tertiary care centre, it receives patients from other hospitals in the country. Moreover, herein a comprehensive microbiological and molecular characterization of carbapenem-resistant *K. pneumoniae* clinical strains was performed, contributing updated data on the genomic epidemiology, resistome, and virulome. Future studies should expand both the collection of strains analysed by WGS and the number of hospitals involved, in order to provide a more complete picture of the molecular patterns of *K. pneumoniae* in Portugal.

## 5. Conclusions

In conclusion, we reported a molecular epidemiology shift in Portugal and an uncommon clone detection compared to Europe (where CG15 and CG258 are still dominant). The high-risk clones ST13, ST17, ST147, and ST307 were the most predominant among the carbapenem-resistant *K. pneumoniae* strains studied. Furthermore, we described *K. pneumoniae* strains with a high prevalence of siderophores, suggesting the emergence of hypervirulent strains in Portugal. These results emphasized the relevance of performing continuous molecular surveillance, especially through WGS.

## Figures and Tables

**Table 1:** Disk diffusion test results showing distribution for 437 clinical *K. pneumoniae* strains.

Antibiotic Tested	AST Dose (μg)	Interpretation ^a^			Susceptibility Rate (%)
		S	I	R	
Penicillins: Amoxicillin–clavulanic acid	20/10	2	0	435	0.5%
Cephalosporins: Cefoxitin Cefotaxime Ceftazidime Ceftazidime–avibactam	30 5 10 10/4	97 32 22 436	0 7 11 0	340 398 404 1	22.2% 8.9% 7.6% 99.8%
Carbapenems: Imipenem Ertapenem Meropenem	10 10 10	72 17 42	50 0 128	315 420 267	27.9% 3.9% 38.9%
Monobactam: Aztreonam	30	24	9	404	7.6%
Fluoroquinolone: Ciprofloxacin	5	90	41	306	30.0%
Aminoglycoside: Gentamicin	10	113	0	324	25.9%
Tetracycline: Tigecycline	15	306	0	131	70.0%

^a^ Following European Committee on Antimicrobial Susceptibility Testing (EUCAST) breakpoints (version 11.0, 2021; available at https://eucast.org/clinical_breakpoints; accessed on 20 December 2021). AST—antimicrobial susceptibility test; S—susceptible; I—susceptible, increased exposure; R—resistant.

**Table 2:** MLST profile, hypermucoviscosity phenotype, resistance and virulence determinants, porin mutation, and plasmid replicons of carbapenem-resistant *K. pneumoniae* strains.

ID FMUL	MLST	Hypermucoviscosity Phenotype	Resistance Profile			Virulence Profile		K Locus	O Locus	OmpK Mutations	Plasmid Replicons
			Carbapenemases	β-Lactamases	Other Resistance Genes	Yersiniabactin	Colibactin				
5	231	no	KPC-3	OXA-9	aac(3)-IId;aadA;aadA16;strA;strB; catII.2; arr-3; sul1;sul2; tet(D); dfrA1;dfrA14*;dfrA27; GyrA-83I;ParC-80I	*ybt 9; ICEKp3*	*-*	KL51	O1v2	*-*	ColKP3; IncFIA(HI1); IncFIB(K)(pCAV1099-114); IncR; IncX3
17	348	no	KPC-3; OXA-181	OXA-9; TEM-1D; SHV-11	*aac(6′)-Ib’;strA;strB; qnrB1;qnrS1;* *sul2; dfrA14*	*ybt* unknown	*-*	KL62	O1v1	*OmpK36-0%*	ColKP3; IncFIA(HI1); IncFIB(K)(pCAV1099-114); IncFII(K); IncR; IncX3
20	147	no	OXA-181	TEM-1D; CTX-M-15; SHV-1	*aac(6′)-Ib-cr;aadA16;strA;strB; qnrS1; arr-3; sul1;sul2; dfrA27; GyrA-83I;ParC-80I*	*ybt 16; ICEKp12*	*-*	KL64	O2v1	*-*	FIA(pBK30683); FII(pBK30683); IncFIB(K); IncFII(K)
22	13	no	KPC-3	OXA-9; TEM-1D; SHV-1	*aac(6′)-Ib’;aadA;strA;strB; sul2; dfrA14*	*ybt 17; ICEKp10*	*clb 3*	KL3	O1v2	*-*	ColKP3; IncFIA(HI1); IncFIB(K)(pCAV1099-114); IncR; IncX3
24	17	no	OXA-181	OXA-1; CTX-M-15; SHV-11	*aac(3)-IIa;aac(6′)-Ib-cr;aadA16;strB; qnrB4;qnrS1; catII.2; arr-3; sul1;sul2; dfrA27*	*-*	*-*	KL25	O5	*-*	ColRNAI; FIA(pBK30683); FII(pBK30683)
26	147	no	OXA-181	TEM-1D; CTX-M-15	*aac(6′)-Ib-cr;aadA16;strA;strB; qnrS1; arr-3; sul1;sul2; dfrA27; GyrA-83I;ParC-80I*	*ybt 16; ICEKp12*	*-*	KL64	O2v1	*-*	ColRNAI; FIA(pBK30683); FII(pBK30683)
27	17	no	OXA-181	TEM-1D; CTX-M-15; SHV-1	*aac(6′)-Ib-cr;aadA16;strA;strB; qnrS1; arr-3; sul1;sul2; tet(D); dfrA14;dfrA27*	*ybt 16; ICEKp12*	*-*	KL25	O5	*-*	ColRNAI; FIA(pBK30683); FII(pBK30683)
43	17	no	OXA-181	SHV-11	*aac(6′)-Ib-cr;aadA16;strA;strB; catII.2; arr-3; sul1;sul2; dfrA27*	*ybt 16; ICEKp12*	*-*	KL25	O5	*-*	ColKP3, IncFIA(HI1), IncFIB(K)(pCAV1099-114), IncM1, IncR, IncX3
45	17	no	OXA-181	TEM-1D; CTX-M-15; SHV-11	*aac(3)-IIa;aac(6′)-Ib-cr;aadA16;strB; qnrS1; mphA; catII.2; arr-3; sul1;sul2; dfrA27*	*ybt 16; ICEKp12*	*-*	KL25	O5	*-*	IncFIB(K); IncFIB(pKPHS1); IncFII(K); IncX3
48	17	no	OXA-181	SHV-11	*aac(3)-IIa;aac(6′)-Ib-cr;aadA16;strB; qnrS1; catII.2; arr-3; sul1;sul2; dfrA27*	*ybt 16; ICEKp12*	*-*	KL25	O5	*-*	ColRNAI; FIA(pBK30683); FII(pBK30683)
60	34	no	KPC-3	TEM-1D; SHV-26	*strA;strB; sul2; dfrA14*	*-*	*-*	KL153	O1v2	*-*	IncFIB(K); IncFIB(pQil); IncFII(K); IncX3
71	17	no	OXA-181	OXA-1; TEM-1D; CTX-M-15; SHV-11	*aac(3)-IIa;aac(6′)-Ib-cr;aadA16;strB; qnrS1; mphA; catII.2; arr-3; sul1;sul2; dfrA27*	*ybt 16; ICEKp12*	*-*	KL25	O5	*-*	IncC; IncFIB(K)(pCAV1099-114); IncFII(K)
81	37	no	KPC-3	OXA-9; TEM-1D; SHV-11	*aac(6′)-Ib’;aadA;strA;strB; sul2; dfrA14*	*ybt 10; ICEKp4*	*-*	KL14	O3b	*-*	IncFIB(K); IncFII(K)
92	13	yes	KPC-3	OXA-9; TEM-1D; SHV-1	*aac(6′)-Ib’;aadA;strA;strB; sul2; dfrA14; GyrA-87N*	*ybt 17; ICEKp10*	*clb 3*	KL3	O1v2	*OmpK35-70%*	Col440I; Col440II; FIA(pBK30683); FII(pBK30683); IncFIA(HI1); IncFIB(K); IncR
99	13	yes	KPC-3	OXA-9; TEM-1D; SHV-1	*aac(6′)-Ib’;aadA;strA;strB; sul2; dfrA14; GyrA-87N*	*ybt 17; ICEKp10*	*clb 3*	KL3	O1v2	*OmpK35-70%*	ColRNAI; FII(pBK30683); IncFIB(K); IncFII(K)
101	323	no	KPC-3	OXA-1; OXA-9; TEM-1D; SHV-1	*aac(3)-IIa;aadA;strA;strB; qnrB1; sul2; tet(A); dfrA14; GyrA-83F*	*-*	*-*	KL21	O3b	*-*	FIA(pBK30683), FII(pBK30683), IncFIB(K), IncFII(K)
102	392	no	-	OXA-1; TEM-1D; CTX-M-15; SHV-11	*aac(3)-IIa;aac(6′)-Ib-cr;strA;strB; qnrB1; sul2; tet(A); dfrA14; GyrA-83I;ParC-80I*	*-*	*-*	KL27	O4	*OmpK35-58%;* *OmpK36-0%*	IncFIB(K), IncFII(K)
116	147	no	OXA-181	TEM-1D; CTX-M-15; SHV-1	*aac(6′)-Ib-cr;aadA16;strA;strB; qnrS1; arr-3; sul1;sul2; dfrA27; GyrA-83I;ParC-80I*	*ybt 16; ICEKp12*	*-*	KL64	O2v1	*-*	IncFIA(HI1); IncFIB(K); IncR
122	13	yes	KPC-3	OXA-9; TEM-1D; SHV-1	*aac(6′)-Ib’;aadA;strA;strB; sul2; dfrA14; GyrA-87N*	*ybt 17; ICEKp10*	*clb 3*	KL3	O1v2	*OmpK35-70%*	IncFIA(HI1); IncFIB(K); IncR
127	29	yes	OXA-181	-	*qnrS1; tet(D)*	*ybt 10; ICEKp4*	*-*	KL54	O1v2	*-*	IncFIA(HI1); IncFIB(K); IncR
132	37	no	KPC-3	OXA-9; TEM-1D; SHV-11	*aac(6′)-Ib’;aadA;strA;strB; sul2; dfrA14*	*ybt 10; ICEKp4*	*-*	KL14	O3b	*-*	ColRNAI; FIA(pBK30683); FII(pBK30683); IncFIB(K); IncFII(K); IncR
181	13	no	KPC-3	OXA-9; TEM-1D; CTX-M-15; SHV-1	*aac(6′)-Ib’; aadA; aadA5; strA;strB; qnrS1; mphA; sul1;sul2; tet(D); dfrA14;dfrA17*	*ybt 9; ICEKp3*	*-*	KL3	O1v2	*-*	IncFIB(K); IncFII(K)
184	348	no	KPC-3	OXA-9; TEM-1D; SHV-11	*strA;strB; qnrB1; sul2; dfrA14*	*ybt* unknown	*-*	unknown	O1v1	*-*	Col(pHAD28); FIA(pBK30683); FII(pBK30683); IncFIB(K); IncFII(K); IncFII(K); IncX3
224	39	no	NDM-1	OXA-1; OXA-10; TEM-1D; CTX-M-15; SHV-11	*aac(3)-IIa;aac(6′)-Ib-cr;aadA;aph3-Ia;armA;strA;strB; qnrB1;qnrS1; mphE;msrE; catII.2;cmlA5; arr-2; sul2; tet(A); dfrA14;dfrA7*	*ybt 15; ICEKp11*	*-*	KL62	O1v2	*-*	IncFIA(HI1); IncFIB(K); IncL; IncR
233	307	yes	-	OXA-1; TEM-1D; CTX-M-15; SHV-28	*aac(3)-IIa;aac(6′)-Ib-cr;strA;strB; qnrB1; sul2; tet(A); dfrA14; GyrA-83I;ParC-80I*	*ybt 9; ICEKp3*	*-*	KL102	O2v2	*OmpK36-75%*	FIA(pBK30683); FII(pBK30683); IncM1
245	307	yes	KPC-3	OXA-1; TEM-1D; CTX-M-15; SHV-28	aac(3)-IIa;aac(6′)-Ib-cr;strA;strB; qnrB1; sul2; tet(A); dfrA14*; GyrA-83I;ParC-80I	*ybt 9; ICEKp3*	*-*	KL102	O2v2	*OmpK36-75%*	Col(pHAD28); IncFIB(K); IncFIB(pKPHS1); IncFIB(pNDM-Mar); IncFII(Yp); IncL
247	307	yes	-	OXA-1; TEM-1D; CTX-M-15; SHV-28	*aac(3)-IIa;aac(6′)-Ib-cr;strA;strB; qnrB1; sul2; tet(A); dfrA14; GyrA-83I;ParC-80I*	*ybt 9; ICEKp3*	*-*	KL102	O2v2	*OmpK36-75%*	Col(pHAD28); IncFIB(K); IncFIB(pKPHS1); IncFIB(pNDM-Mar); IncFII(Yp); IncL
248	13	no	KPC-3	TEM-1D; SHV-1	*strA;strB; catA4;floR; sul2; tet(D);tet(J); dfrA1;dfrA14*	*ybt 17; ICEKp10*	*clb 3*	KL19	O1v2	*-*	IncFIB(K); IncFIB(pKPHS1); IncFIB(pNDM-Mar); IncFII(Yp); IncL
257	29	no	GES-5	BEL-1; SHV-187	*aac(6′)-Ib4*	*ybt 17; ICEKp10*	*clb 3*	KL30	O1v2	*-*	IncFIA(HI1); IncFIB(K); IncR; ColRNAI
274	307	yes	-	OXA-1; TEM-1D; CTX-M-15; SHV-28	*aac(3)-IIa^;aac(6′)-Ib-cr;strA^;strB; qnrB1^; sul2; tet(A); dfrA14; GyrA-83I;ParC-80I*	*ybt 9; ICEKp3*	*-*	KL102	O2v2	*OmpK36-75%*	FII(pBK30683); IncFIB(K); IncFII(K); IncR
296	147	no	OXA-181	TEM-1D; CTX-M-15	*aac(6′)-Ib-cr;aadA16;strA;strB; qnrS1; arr-3; sul1;sul2; dfrA27; GyrA-83I;ParC-80I*	*ybt 16; ICEKp12*	*-*	KL64	O2v1	*-*	IncFIB(K), IncFIB(pKPHS1), IncFII(K), IncX3
345	5840	no	KPC-3	OXA-9; TEM-1D; SHV-1	*aac(6′)-Ib’;aadA;strA;strB; sul2; dfrA14*	*ybt 10; ICEKp4*	*-*	KL5	O3b	*-*	ColKP3; ColRNAI; FIA(pBK30683); FII(pBK30683); IncFIB(K); IncFII(K); IncR; IncX3
363	3031	yes	-	SHV-1	*-*	*ybt 10; ICEKp4*	*-*	KL24	O2v1	*-*	IncFIB(K)
443	17	no	OXA-181	OXA-1; SHV-11	*aac(3)-IIa;aac(6′)-Ib-cr;aadA16;strA;strB; qnrS1; arr-3; sul1;sul2; dfrA27*	*ybt 16; ICEKp12*	*-*	KL25	O5	*-*	ColKP3; IncFIA(HI1); IncFIB(K) (pCAV1099-114); IncFII(K); IncR; IncX3
447	231	no	-	OXA-1; TEM-1D; CTX-M-15; SHV-1	*aac(3)-IIa;aac(6′)-Ib-cr;aadA2;aph3-Ia;strA;strB; qnrB1; mphA; sul1;sul2; dfrA12;dfrA14; GyrA-83I;ParC-80I*	*ybt 10; ICEKp4*	*-*	KL51	O1v2	*-*	ColKP3; ColRNAI; FIA(pBK30683); FII(pBK30683); IncFIB(K); IncFII(K); IncR; IncX3
449	13	no	KPC-3; OXA-181	TEM-1D; SHV-1	*strA;strB; qnrS1; sul2; tet(D); dfrA14*	*ybt 17; ICEKp10*	*clb 3*	KL19	O1v2	*-*	IncFIB(K); IncFIB(pKPHS1); IncFII(K); IncR; IncX3
451	17	no	OXA-181	OXA-1; TEM-1D; CTX-M-15; SHV-11	*aac(3)-IIa;aac(6′)-Ib-cr;aadA16;strA;strB; qnrS1; mphA; arr-3; sul1; dfrA27*	*ybt 16; ICEKp12*	*-*	KL25	O5	*-*	ColKP3; IncFIA(HI1); IncFIB(K)(pCAV1099-114); IncR; IncX3
460	13	yes	KPC-3; OXA-181	TEM-1D; SHV-1	*strA;strB; qnrS1; sul2; tet(D); dfrA14*	*ybt 17; ICEKp10*	*clb 3*	KL19	O1v2	*-*	IncFIA(HI1); IncFIB(K); IncR
462	17	no	OXA-181	OXA-1; TEM-1D; CTX-M-15	*aac(3)-IIa;aac(6′)-Ib-cr;aadA16;aadA;strA;strB; qnrS1; mphA; arr-3; sul1;sul2; dfrA27*	*ybt 16; ICEKp12*	*-*	KL25	O5	*-*	ColRNAI; FIA(pBK30683); FII(pBK30683); IncFIB(K); IncFIB(pKPHS1); IncFII(K); IncR;
463	17	no	OXA-181	OXA-1; TEM-1D; CTX-M-15; SHV-11	*aac(3)-IIa;aac(6′)-Ib-cr;aadA16;strB; qnrS1; mphA; arr-3; sul1;sul2; dfrA27*	*ybt 16; ICEKp12*	*-*	KL25	O5	*-*	IncFIB(K); IncFIB(pKPHS1); IncFII(K); IncR
465	17	no	OXA-181	OXA-1; TEM-1D;CTX-M-15; SHV-11	*aac(3)-IIa;aac(6′)-Ib-cr;aadA16;strB; qnrS1; mphA; arr-3; sul1;sul2; dfrA27*	*ybt 16; ICEKp12*	*-*	KL25	O5	*-*	ColKP3; IncFIA(HI1); IncFIB(K)(pCAV1099-114); IncFII(K); IncR; IncX3
467	45	no	KPC-3	TEM-1D; SHV-1	*strA;strB; sul2; tet(D); dfrA14*	*ybt 10; ICEKp4*	*-*	KL62	O2v1	*-*	IncFIB(K); IncFIB(pKPHS1); IncFII(K); IncX3
470	45	no	-	OXA-1; TEM-1D; CTX-M-15; SHV-1	*aac(3)-IIa;aac(6′)-Ib-cr;strA;strB; qnrB1; sul2; dfrA14*	*ybt 10; ICEKp4*	*-*	KL62	O2v1	*-*	ColRNAI; FIA(pBK30683); FII(pBK30683); IncFIB(K)
471	17	no	OXA-181	TEM-1; CTX-M-15; SHV-11	*aac(3)-IIa;aac(6′)-Ib-cr;aadA16;strA;strB; qnrS1; mphA; arr-3; sul1; dfrA27*	*ybt 16; ICEKp12*	*-*	KL25	O5	*-*	ColRNAI; FIA(pBK30683); FII(pBK30683); IncFIB(K)
473	45	no	OXA-181	OXA-1; TEM-1D;CTX-M-15; SHV-1	*aac(3)-IIa;aac(6′)-Ib-cr;strA;strB; qnrB1;qnrS1; sul2; tet(D); dfrA14*	*ybt 10; ICEKp4*	*-*	KL62	O2v1	*-*	ColKP3; ColRNAI; FIA(pBK30683); FII(pBK30683); IncFIB(K); IncFII(K); IncR; IncX3
476	17	no	OXA-181	OXA-1; TEM-1D; CTX-M-15; SHV-11	*aac(3)-IIa;aac(6′)-Ib-cr;aadA16;strA;strB; qnrS1; mphA; arr-3; sul1;sul2; dfrA27*	*ybt 16; ICEKp12*	*-*	KL25	O5	*-*	IncFIB(K); IncFII(K)
498	13	yes	KPC-3; OXA-181	TEM-1D; SHV-1	*strA;strB; qnrS1; sul2; tet(D); dfrA14*	*ybt 17; ICEKp10*	*clb 3*	KL19	O1v2	*-*	IncFIB(K); IncFII
499	307	no	-	OXA-1; TEM-1D; CTX-M-15; SHV-28	*aac(3)-IIa;aac(6′)-Ib-cr;strA;strB; qnrB1; sul2; tet(A); dfrA14; GyrA-83I;ParC-80I*	*ybt 9; ICEKp3*	*-*	KL102	O2v2	*OmpK35-30%*	ColRNAI; FIA(pBK30683); FII(pBK30683); IncFIB(K); IncFII(K); IncR
500	307	no	-	OXA-1; TEM-1D; CTX-M-15; SHV-28	*aac(3)-IIa;aac(6′)-Ib-cr;strA;strB; qnrB1; sul2; tet(A); dfrA14; GyrA-83I;ParC-80I*	*ybt 9; ICEKp3*	*-*	KL102	O2v2	*OmpK35-30%*	ColKP3; IncFIA(HI1); IncFIB(K) (pCAV1099-114); IncR; IncX3
501	3031	no	-	CTX-M-15	*aadA5; qnrS1; mphA; sul1; dfrA17*	*ybt 10; ICEKp4*	*-*	KL24	O2v1	*OmpK36-85%*	Col156; ColKP3; IncFIA(HI1); IncFIB(AP001918); IncFIB(K)(pCAV1099-114); IncFII; IncR; IncX3
502	147	no	OXA-181	TEM-1D; CTX-M-15	*aac(6′)-Ib-cr;aadA16;strA;strB; qnrS1; arr-3; sul1;sul2; dfrA27*	*ybt 16; ICEKp12*	*-*	KL64	O2v1	*-*	IncFIB(K)(pCAV1099-114); IncFIB(pQil); IncFII(K)
516	13	no	KPC-3	TEM-1D^	*strA;strB; sul2; tet(D); dfrA14*	*ybt 17; ICEKp10*	*clb 3*	-	O1/O2v2	*-*	Col440II; ColRNAI; FIA(pBK30683); FII(pBK30683); IncFIB(K)
517	13	no	KPC-3	TEM-1D; SHV-1	*strA;strB; sul2; tet(D); dfrA14*	*-*	*-*	-	O1/O2v2	*-*	IncFIB(K)(pCAV1099-114); IncFIB(pQil); IncFII(K)
522	661	no	-	OXA-10; TEM-1D; SHV-27	*aac(6′)-Ib;aadA;aadA2;strA;strB; qnrS1; mphA; sul1;sul2; tet(A); dfrA12;dfrA14*	*ybt 14; ICEKp5*	*-*	KL27	O2v2	*-*	ColKP3; ColRNAI; FIA(pBK30683); FII(pBK30683); IncFIB(K); IncFII(K); IncR; IncX3
523	13	no	KPC-3	TEM-1D	*strA^;strB; sul2; tet(D); dfrA14*	*ybt 17; ICEKp10*	*clb 3*	-	O1v2	*-*	IncFIB(K); IncFII(K)
533	661	no	-	OXA-10; TEM-1D; SHV-27	*aac(6′)-Ib’;aadA;aadA2;strA;str; qnrS1; mphA; sul1;sul2; tet(A); dfrA12;dfrA14*	*ybt 14; ICEKp5?*	*-*	KL27	O2v2	*-*	IncFIB(K); IncFIB(pKPHS1); IncFII(K); IncN
535	13	no	KPC-3; OXA-181	TEM-1D; SHV-1	strA;strB; qnrS1; sul2; tet(D); dfrA14*	*ybt 17; ICEKp10*	*clb 3*	-	O1v2	*-*	Col440I; FIA(pBK30683); FII(pBK30683); IncFII(pCoo); IncN
548	307	no	-	OXA-1; TEM-1; CTX-M-15; SHV-28	*aac(3)-IIa;aac(6′)-Ib-cr;strA;strB; qnrB1; sul2; tet(A); dfrA14*	*ybt 9; ICEKp3*	*-*	KL102	O2v2	*-*	Col156; IncFIB(AP001918); IncFIB(pKPHS1); IncFII; IncN
584	17	no	OXA-181	OXA-1; CTX-M-15; SHV-11	*aac(3)-IIa;aac(6′)-Ib-cr;aadA16;strA;strB; qnrS1; mphA; arr-3; sul1;sul2; dfrA27*	*ybt 16; ICEKp12*	*-*	KL25	O5	*-*	FIA(pBK30683); FII(pBK30683)
592	17	no	OXA-181	TEM-1D; CTX-M-15; SHV-11	*aac(3)-IIa;aac(6′)-Ib-cr;aadA16;strB; qnrS1; mphA; arr-3; sul1;sul2; dfrA27*	*ybt 16; ICEKp12*	*-*	KL25	O5	*-*	Col440I; FIA(pBK30683); FII(pBK30683); IncR
595	485	no	KPC-3	TEM-1D; SHV-1	*aadA;strA^;strB; sul2; dfrA14*	*ybt 10; ICEKp4*	*-*	-	OL101	*-*	Col440II; FIA(pBK30683); FII(pBK30683); IncFIB(K)(pCAV1099-114)
596	147	no	OXA-181	TEM-1D; CTX-M-15-; SHV-1	*aac(6′)-Ib-cr;aadA16;strA^;strB; qnrS1; arr-3; sul1;sul2; dfrA27*	*ybt 16; ICEKp12*	*-*	KL64	O2v1	*-*	IncFIB(K); IncFII(K)
648	13	no	KPC-3; OXA-48	TEM-1D; SHV-1	*strA^;strB; sul2; tet(D); dfrA14*	*ybt 17; ICEKp10*	*clb 3*	KL19	O1v2	*-*	ColRNAI, FIA(pBK30683), FII(pBK30683), IncFIB(K), IncL
679	307	no	-	SHV-28	*GyrA-83I;ParC-80I*	*ybt 9; ICEKp3*	*-*	KL102	O2v2	*-*	-
680	231	no	KPC-3	-	*aac(6′)-Ib; dfrA14; GyrA-83I;ParC-80I*	*ybt 9; ICEKp3*	*-*	KL51	O1/O2v2	*-*	Col440I, Col440II, FIA(pBK30683), FII(pBK30683), IncFIB(K), IncFII(K), IncR
681	13	no	KPC-3	TEM-1D	*strA;strB; sul2; tet(D); dfrA14*	*ybt 17; ICEKp10*	*clb 3*	KL19	O1v2	*-*	ColRNAI, FIA(pBK30683), FII(pBK30683), IncFIB(K)
683	13	no	KPC-3	TEM-1D; SHV-1	*strA;strB; sul2; dfrA14; GyrA-87N*	*ybt 17; ICEKp10*	*clb 3*	KL3	O1v2	*-*	ColRNAI, FIA(pBK30683), FII(pBK30683)
685	13	no	KPC-3	TEM-1D; SHV-1	*strA;strB; sul2; tet(D); dfrA14*	*ybt 17; ICEKp10*	*clb 3*	KL19	O1v2	*-*	ColRNAI, FIA(pBK30683), FII(pBK30683), IncFIB(K)

## Data Availability

Not applicable.

## Supplementary Materials

The following are available online at https://www.mdpi.com/article/10.3390/microorganisms10020416/s1, Table S1: Information about the assembly of the 68 carbapenem-resistant *K. pneumoniae* sequenced by WGS and *de novo* assembled genomes.
