# Supplementary material for: Whole-Genome Sequencing Enables Molecular Characterization of Non-Clonal Group 258 High-Risk Clones (ST13, ST17, ST147 and ST307) among Carbapenem-Resistant Klebsiella pneumoniae from a Tertiary University Hospital Centre in Portugal
Source: Microorganisms. 2022 Feb 11;10(2):416. doi: 10.3390/microorganisms10020416 (PMC8875758; doi:10.3390/microorganisms10020416)
Supplement: Supplementary file 1 [file microorganisms-10-00416-s001.zip › microorganisms-1551505 - supplementary.pdf]

Whole-Genome Sequencing Enables Molecular Characterization of Non-Clonal Group 258 High-Risk Clones (ST13, ST17, ST147 and ST307) Among Carbapenem-Resistant *Klebsiella pneumoniae* From a Tertiary University Hospital Centre in Portugal

Mendes, G.; Ramalho, J.F.; Bruschy-Fonseca, A.; Lito, L.; Duarte, A.; Melo-Cristino, J.; Caneiras, C.

Table S1. Information about the assembly of the 68 carbapenem-resistant *K. pneumoniae* sequenced by WGS and *de novo* assembled genomes.

Identification (ID) FMUL	Number of Contigs	Total contig length	Largest contig	N50	Average coverage
5	125	5611042	554205	298305	256.61
17	113	5719986	584048	199665	187.49
20	76	5706460	915546	286255	136.86
22	42	5491341	740786	405384	225.80
24	104	5450169	487855	214006	216.60
26	77	5705687	915545	286255	197.25
27	103	5637389	658766	263369	191.93
43	100	5 604 241	610066	195765	192.23
45	107	5617887	552923	194248	192.09
48	90	5545841	793180	359130	201.36
60	109	5615414	1037085	259885	256.61
71	95	5512395	792761	268298	226.19
81	96	5536904	863147	178236	226.03
92	46	5489913	923505	405414	243.02
99	49	5489507	924021	348058	207.11
101	102	5 580 661	858711	307551	221.03
102	82	5 551 519	604608	214444	212.58
116	78	5684618	708530	243585	219.15
122	46	5489302	923934	363628	213.91
127	87	5580241	978953	376198	227.76
132	81	5 539 418	1028473	294442	306.07
181	108	5 719 355	692567	309497	317.55
184	98	5 758 538	705745	216165	280.11
224	98	5724103	433151	260519	227.87
233	79	5 695 553	530336	226198	276.76
245	233	5 839 418	685269	198011	280.73
247	77	5696342	529808	226209	114.96
248	177	9 913 695	730956	225816	176.11
257	78	5518529	727932	338209	541.27
274	73	5 695 004	530340	230677	372.12
296	83	5 691 504	779964	207802	335.35
345	51	5 443 567	681408	293448	339.12
363	63	5 452 377	385894	247705	215.44

Identification (ID) FMUL	Number of Contigs	Total contig length	Largest contig	N50	Average coverage
443	92	5542756	718515	188012	248.67
447	73	5 506 662	951136	292368	299.04
449	111	5 853 655	636239	253169	337.40
451	100	5 621 958	792956	194250	357.03
460	113	5819352	656250	253401	278.37
462	109	5656818	792478	263369	197.63
463	104	5627251	793391	359320	227.14
465	104	5624043	792801	184560	202.03
467	128	5689145	500882	219420	243.83
470	107	5611340	750180	270645	306.01
471	74	5484948	727516	240748	293.41
473	116	5672086	493556	268795	359.93
476	91	5 555 088	610160	268289	279.68
498	117	5 828 917	630406	253191	279.68
499	75	5 689 862	576250	229813	385.79
500	75	5 694 730	530271	221521	309.13
501	74	5 629 248	658574	247536	330.53
502	87	5691206	708559	181455	252.18
516	98	5702226	525355	249559	325.68
517	105	5569185	516793	169483	285.81
522	61	5444295	741641	244598	301.51
523	93	5703955	630384	255323	233.1
533	61	5614083	553264	296524	220.35
535	114	5830252	533496	295588	319.28
548	75	5666757	576248	226159	256.02
584	102	5547028	546112	178301	257.18
592	89	5527889	535306	194812	266.19
595	54	5356080	529913	268978	283.28
596	83	5689842	610673	206447	238.86
648	102	5 800 512	610489	315125	209.96
679	59	5 503 955	806216	301075	186.85
680	88	5 581 723	571899	351014	228.94
681	104	5 672 768	550247	233325	212.63
683	43	5 487 624	540594	329632	253.51
685	100	5 674 813	547490	238159	244.10
